# *In situ* generation of turbostratic nickel hydroxide as a nanozyme for salivary glucose sensor†

**DOI:** 10.1039/d4ra03559c

**Published:** 2024-07-09

**Authors:** Priya Pathmanathan, A. Gomathi, Asha Ramesh, Ch. Subrahmanyam

**Affiliations:** a Department of Chemistry, Mahindra University Hyderabad-500043 India gomathi.anandhanatarajan@mahindrauniversity.edu.in; b Department of Chemistry, Indian Institute of Technology Hyderabad-502285 India

## Abstract

Among the 3d-transition metal hydroxide series, nickel hydroxide is a well-studied electroactive catalyst. In particular, nickel hydroxide and its composite materials are well-suited for non-enzymatic glucose sensing. The electrocatalytic efficiency of nickel hydroxide is attributed to the thickness or to be precise, the thinness of the electroactive layer. Herein, we have successfully prepared metallic nickel@nickel hydroxide nanosheets through a straightforward one-pot solvothermal method. We were able to electrochemically generate a highly sensitive α-Ni(OH)_2_ on the nanosheets. The dynamic generation and synergy between α- and β-Ni(OH)_2_, imparts a glucose oxidase enzyme-like ability to the catalyst. Our proposed nickel nanozyme exhibits a good sensitivity of 683 μA mM^−1^ cm^−2^ for glucose. The sensor operates in the range of 0.001–3.1 mM, with a lower limit of detection (LOD) of 9.1 μM and exhibits a response time of ≈00.1 s. Nickel-nanozyme demonstrated better selectivity for glucose in the presence of interfering compounds. Notably, the sensor does not suffer from an interfering oxygen evolution reaction. This greatly improves sensitivity in glucose detection in lower concentrations making the sensor viable to measure salivary glucose levels. In this study, we demonstrate that our sensor can detect glucose in human saliva. The real sample analysis was carried out with saliva samples from three healthy human volunteers and one prediabetic volunteer. Our proposed sensor measurements show excellent agreement with calculated salivary glucose levels with 98% accuracy in sensing glucose in real saliva samples.

## Abstract 2

Turbostratic nickel hydroxide as a salivary glucose sensor.

## Graphical abstract

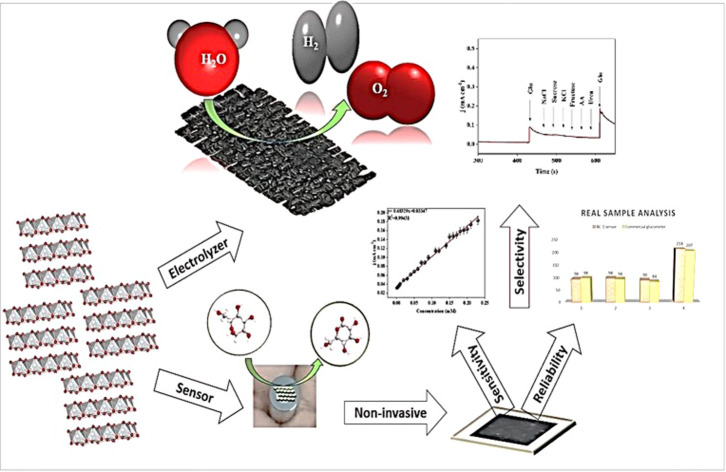

## Introduction

Diabetes mellitus is a significant global health concern worldwide, giving rise to countless complications as a co-morbid health condition.^1^ According to a report by Lin *et al.*,^2^ the burden on the population due to diabetics has been steadily increasing since 2018. Thus, to frequently monitor glucose levels, it is necessary to fabricate an accurate clinical diagnostic device for glucose detection.^3^ Glucose levels can be detected by either of the two types namely, enzymatic and non-enzymatic sensors.^4,5^ In enzymatic glucose sensing, glucose oxidase or glucose dehydrogenase oxidizes glucose molecules. For each molecule of glucose oxidized by the enzyme, one electron is generated by the reaction. This electron is sensed by the working electrode and converted into a signal. To the best of our knowledge, most commercial electrochemical sensors are enzymatic glucose sensors, and this type of sensor is preferred due to their high sensitivity and specific selectivity for glucose sensing. But enzyme-based sensors are sensitive to ambient conditions such as pH, temperature, and humidity. Any variation in these parameters could potentially lead to enzyme degradation which in turn affects the stability of enzymatic glucose sensors, thus reducing their lifetime.^6,7^ To address these demerits, significant effort is being undertaken to fabricate non-enzymatic sensors with enhanced selectivity, sensitivity, and durability.^8^

In a non-enzymatic glucose sensor, glucose gets oxidized on a redox-activated metal oxide/hydroxide surface coated on a conducting electrode. This electrode will then be used to sense the electron generated by the oxidation of glucose. Nanomaterials of transition metals (Au,^9,10^ Pt,^9,11^ Ag,^12^ Co,^13^ Cu,^14–16^ Ni^17^),^18–21^ metal oxides^22^ (Co_3_O_4_, NiO, Fe_2_O_3_)^23–28^ and metal hydroxides (Co(OH)_2_, Ni(OH)_2_, Fe(OH)_2_)^29–33^ have been widely studied for their sensing efficiency. Noble metal^34^ based catalysts such as gold,^35^ silver, and platinum are easy to fabricate^19^ and have excellent chemical and physical qualities such as high conductivity and high specific surface area. But these catalysts suffer from high cost and sluggish kinetics that minimize the faradaic current, thus hindering their sensing capacity. In addition to the above demerits, the surface of noble metals is easily contaminated by intermediates such as chloride ions, resulting in a loss of active sites. To circumvent these negative aspects of noble metal-based sensors, non-noble materials based on nickel, copper, cobalt and iron have^22,36–40^ been synthesized in the form of metallic nanoparticles, oxides and hydroxide nanostructures for glucose sensing. Among the various d-block metal compounds, multi-crystal phase, α-Ni(OH)_2_ and β-Ni(OH)_2_,^41–43^ has been studied extensively for its use as a non-enzymatic glucose sensor for its dominant electrocatalytic activity and high stability. Morphology of nickel hydroxide can be tailored to enhance the electrocatalytic activity of nickel hydroxide-based composites. For instance, Pal *et al.*^44^ reported hydrothermal aided design of β-Ni(OH)_2_ and NiO nanorods with the latter showing a better response in sensing d-glucose.^7,44^ Additionally, Ni(OH)_2_@Ni core shell nanochains were prepared by chemical bath deposition (CBD) followed by reductive annealing and electrochemical oxidation. Enhanced activity of nanostructures is attributed to the porous core shell design that enables faster electron transfer and enhanced consumption of active material.^45^ Zhong *et al.*^46^ observed that introduction of defects in nickel hydroxide nanosheets on Ni foam led to better glucose sensor. Electrochemical arrangement of Ni(OH)_2_ on Ni@nitrogen-doped nanodiamond substrate, exhibited thickness-dependent sensing capabilities of the hydroxide layer.^47^ Atomic layer deposition of NiS_*x*_ on carbon cloth, followed by electrochemical generation of Ni(OH)_2_ electrocatalyst, showed superior glucose sensing activity.^29^ The pre-electrocatalyst NiS_*x*_ layer imparts high roughness and in turn imparts superior glucose sensing activity to the hydroxide electrocatalyst. The roughness has been directly proportional to the number of electroactive sites of nickel hydroxide.^29^ Nano petals of nickel hydroxide were formed on Ni/metallic glass substrate by a two-step process. High glucose sensitivity of the material is facilitated by an efficient charge transfer imparted by the sheet like morphology of nickel hydroxide.^48^ Hence, to design and fabricate a superior nickel hydroxide glucose sensor, careful consideration should be focused towards three aspects: morphology of the electrocatalytic material, thickness of electroactive Ni(OH)_2_/NiOOH surface and presence of synergy between conducting metallic surface and electroactive surface to sense the electrons generated by oxidation of glucose.

In this report, we propose an ultra-high-sensitive glucose sensor, Ni@Ni(OH)_2_, a hetero nanostructure, fabricated by one-pot solvothermal synthesis. We show that a highly active nickel hydroxide electrocatalyst is generated electrochemically and there exists a dynamic conversion between two electrochemically active α- and β-Ni(OH)_2_ catalytic phases. The nickel nanozyme exhibits an excellent sensitivity of 683 μA mM^−1^ cm^−2^ for the electro-oxidation of glucose, with a detection range of 0.001 to 3.1 mM, and a low detection limit (LOD) of 9.1 μM.

## Results and discussion

All the nickel-based nanostructures were prepared by one-step solvothermal process. To prepare pure β-Ni(OH)_2_, nickel acetylacetonate was solvothermally treated with 12 M NaOH for 24 hours at 120 °C. This sample has been named as “NC-3”. To form metallic nickel@nickel hydroxide composite nanostructures, hydrazine was added to the reaction medium and heated at 120 °C for two different time durations. The sample obtained after 24 hours of reaction is named as “NC-1”. The sample obtained after 12 hours under same experimental conditions is named as “NC-2”. Based on our rationale, NC-1 will have more metallic nickel than NC-2 as the reaction was carried out for longer duration to make the former sample.

**Fig. 1:**
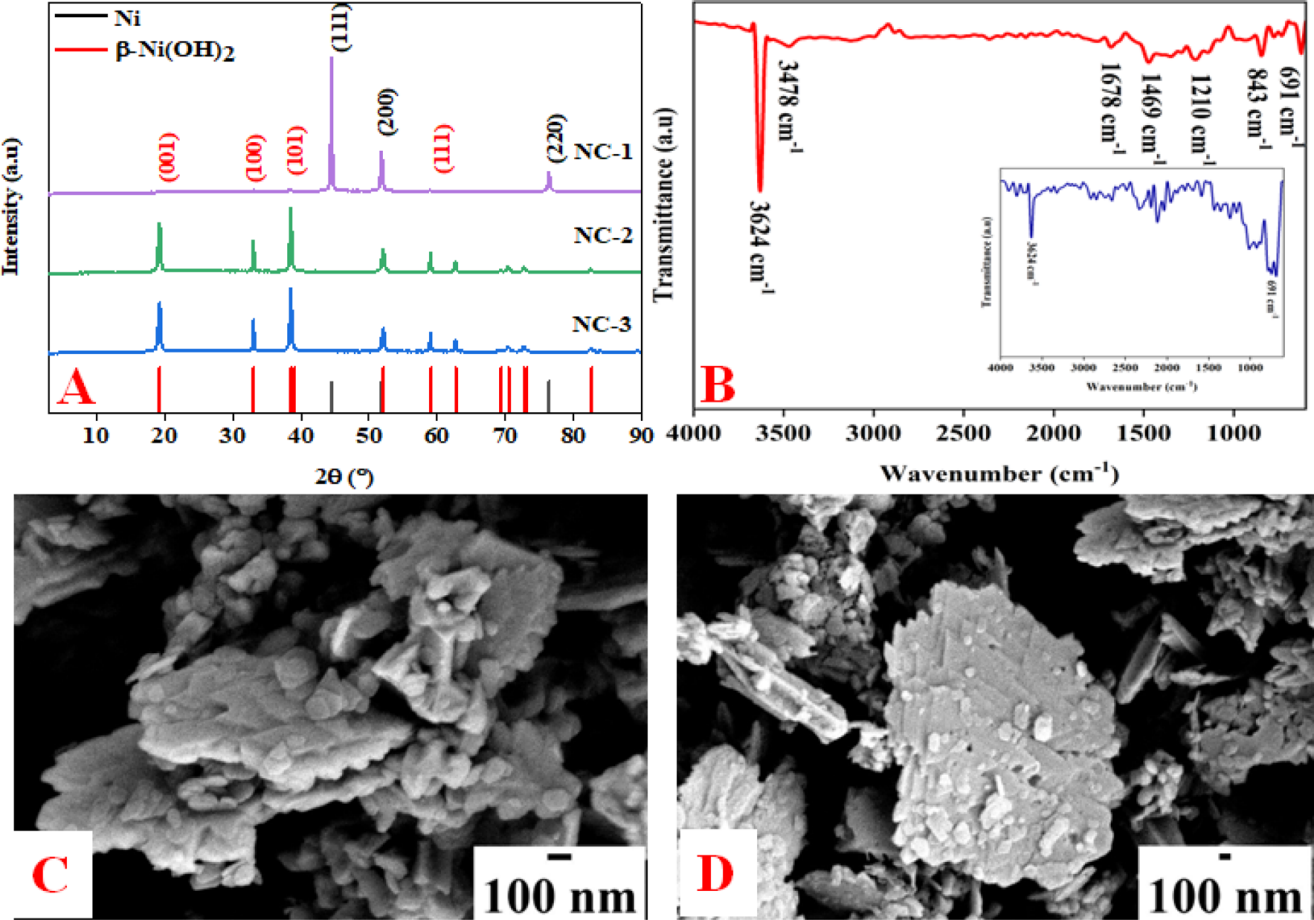
(A) XRD pattern of NC-1, NC-2 and NC-3. (B) IR spectrum of NC-1, NC-2 and NC-3. (C) and (D) FESEM images of NC-3 and NC-1.

**Fig. 2:**
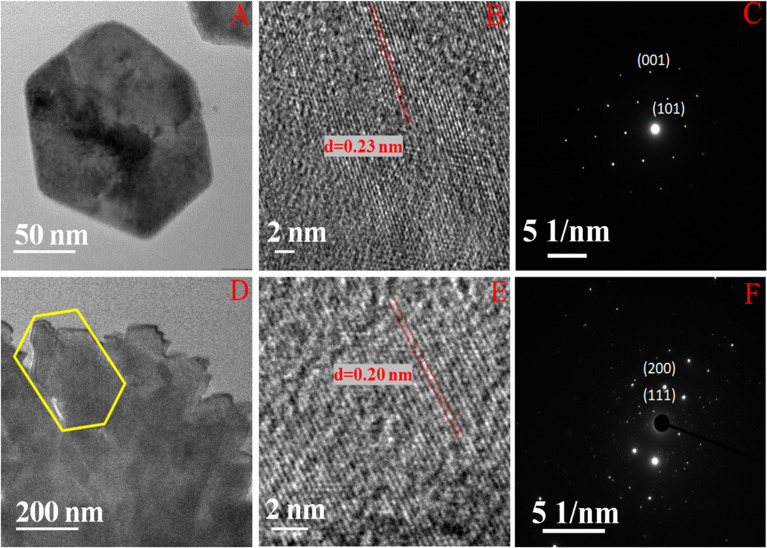
(A) and (B) Indicate TEM and HRTEM images of NC-3. (D) and (E) Indicate TEM and HRTEM images of NC-1. (C) and (F) Indicate the SEAD pattern of NC-3 and NC-1.

**Fig. 3:**
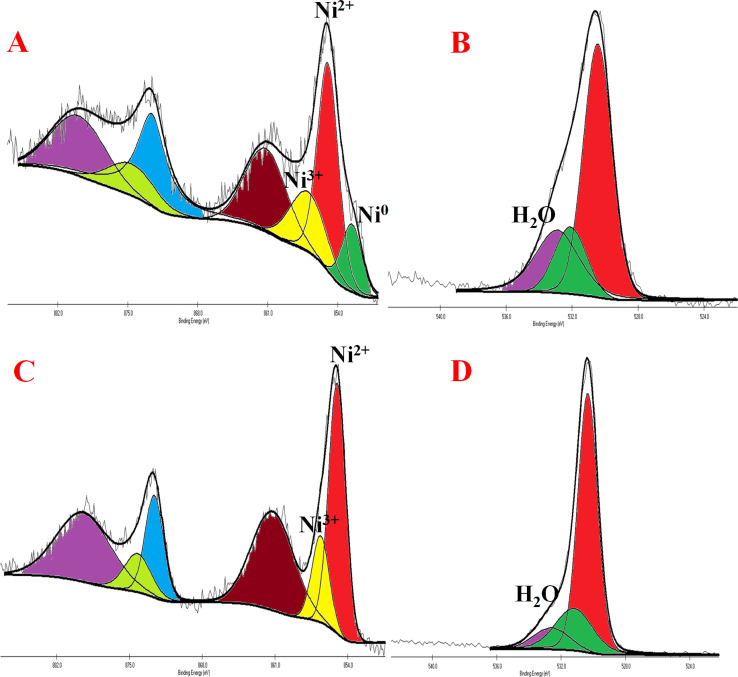
(A) and (C) Indicate high resolution XPS images of NC-3 and NC-1 of Ni 2p peaks (B) and (D) indicate XPS images of NC-3 and NC-1 of O 1s peaks.

Nickel hydroxide crystallizes in two polymorphic structures, turbostratic α-Ni(OH)_2_ and a highly ordered β-Ni(OH)_2_. In Fig. 1(A), we present the X-ray diffraction (XRD) patterns of the compounds listed in Table 1. Sample NC-3, characterized by a hexagonal crystal structure, is identified as pure β-Ni(OH)_2_, with all facets precisely matching the β-Ni(OH)_2_ pattern (ICDD card no. 014-0117). NC-3 exhibits lattice parameters of *a* = 3.126 Å and *c* = 4.605 Å. The XRD patterns of NC-1 and NC-2 display reflections from both metallic nickel (ICDD card no. 004-0850) and β-Ni(OH)_2_. The peaks 44.43°, 51.73°, and 76.23° present both in NC-1 and NC-2 correspond to (111), (200) and (220) facets of metallic nickel. In NC-1, nickel is the major phase with trace amounts of β-Ni(OH)_2_. The ratio of peak intensities of Ni/β-Ni(OH)_2_ in NC-1 is greater than that of NC-2. The preparation of NC-3 is essentially a hydrolysis reaction of a nickel salt in a basic solution under solvothermal conditions. The high molarity of sodium hydroxide at 12 M, facilitates the formation of well-organized highly ordered β-Ni(OH)_2_. In preparation of NC-1 and NC-2, hydrazine hydrate was added to the reaction mixture to reduce nickel hydroxide to metallic nickel. Both NC-1 and NC-2 are created in the same chemical environment, but NC-1 is subjected to a longer reaction duration. From the analysis of XRD patterns, it becomes evident that the hydrolysis of nickel acetylacetonate in the presence of 12 M NaOH results in a highly crystalline β-Ni(OH)_2_. Introduction of hydrazine hydrate to the reaction medium reduces β-Ni(OH)_2_ to metallic nickel. FTIR spectra of as-synthesized compounds is shown in Fig. 1(B). Both NC-3 and NC-1 exhibit a sharp peak at 3626 cm^−1^ corresponding to the non-hydrogen bonded –OH stretching of Ni(OH)_2_.^49^ In NC-3, FTIR peaks at 1455 cm^−1^ and 1210 cm^−1^ indicate the presence of bicarbonate. NC-3 also has peaks around 843 cm^−1^ and 618 cm^−1^ corresponding to interlamellar carbonate and Ni–OH bending vibrational band of β-Ni(OH)_2_ respectively.^50–52^ The broad peak at 3478 cm^−1^ and 1678 cm^−1^ represent the surface adsorbed water molecule. FTIR spectrum of NC-1, shown as an inset in the Fig. 1(B) has peaks corresponding to non-bonded hydroxyl group, but does not show strong signature peaks for intercalated carbonate. β-Ni(OH)_2_ is a layered compound and has the ability to accommodate carbonate ions in-between the layers. When the hydroxide gets reduced to metallic nickel, concentration of adsorbed carbonate is decreased by the loss of layered structure. Fig. 1(C) and (D) depict the field emission scanning electron microscope (FESEM) images of NC-3 and NC-1 respectively. NC-3, pure β-Ni(OH)_2_, has hexagonal sheet-like morphology with average sheet-length of ≈200 nm and sheet-thickness of approximately 50 nm. NC-2 has similar morphology of hexagonal sheets (Fig. S1†) as that of NC-3. Addition of hydrazine hydrate did not impart a drastic change in the morphology of nanostructure. No distinct new structural features were observed in NC-2, as hydrazine hydrate successfully reduced the nickel hydroxide to metallic nickel. Meanwhile, NC-1 shown in Fig. 1(D) has a larger sheet like morphology formed by the agglomeration of smaller sheets. To understand the crystalline nature of hydroxide sheets, we carried out TEM analysis. Fig. 2(A)–(C) show the TEM, HRTEM and SAED images of NC-3. Nickel hydroxide sheets are hexagonal in shape and single crystalline in nature. This is evident from Fig. 2(A), which shows TEM image of a single β-Ni(OH)_2_ hexagonal sheet. The HRTEM image in Fig. 2(B) with a d-spacing value of 0.23 nm, corresponding to the (101) facet of nickel hydroxide, confirms the single crystalline nature of β-Ni(OH)_2_ hexagonal sheet. The SAED pattern in Fig. 2(C) is that of the single sheet shown in Fig. 2(B). The reflections were indexed to the crystal planes of β-nickel hydroxide. Fig. 2(E) and (F) show the TEM and HRTEM images of NC-1, where it can be seen that the morphology of NC-1 is still sheet-like but the sheets are larger than NC-3 and does not have defined edges. The HRTEM image exhibits a d-spacing of 0.2 nm corresponding to (111) facet of metallic nickel. The SEAD pattern (Fig. 2(F)) reflections could be indexed to the planes of nickel. We also carried out X-ray photoelectron spectroscopy (XPS) analysis of NC-3 and NC-1 to understand the surface oxidation state of nickel in the as-synthesized samples. High resolution XPS spectrum of Ni 2p of NC-3, is shown in Fig. 3(A). Two peaks at binding energy (BE) values of 855.4 and 873 eV with a separation of 17.6 eV can be assigned to the spin orbit doublets of nickel, Ni 2p_3/2_ and Ni 2p_1/2_, respectively. Ni 2p_3/2_ peak at 855.4 eV, can be fit into two peaks which can be assigned to two different oxidation states of nickel, Ni^2+^ and Ni^3+^. The higher oxidation arises from the surface oxidation of Ni(OH)_2_. On the other hand, high resolution XPS spectrum of NC-1 in Fig. 3(C) is fit into three peaks with an additional peak at 852.3 eV indicating the presence of metallic nickel, Ni^0^. Formation of metallic nickel can be explained by reduction of Ni(OH)_2_ by the hydrazine present in reaction medium during the synthesis of NC-1. Notably in the NC-1 sample, ratio of peak intensities of Ni^3+^/Ni^2+^ has decreased when compared to NC-3, indicating the reduction of Ni^3+^ by introduction of hydrazine.^53–55^Fig. 3(B) and (D) shows the O 1s XPS spectra of NC-3 and NC-1 respectively. Oxygen XPS peak in both the samples indicate presence of three oxygen species corresponding to lattice oxygen of Ni(OH)_2_, surface hydroxyl oxygen and oxygen contribution from adsorbed water. We observe that NC-3 has less adsorbed surface water compared to NC-1.

**Table 1:** Synthesis conditions and name of the compound prepared in this report

Compound	Hydrazine	Temperature (°C)	Time (h)
NC-1	Yes	120	24
NC-2	Yes	120	12
NC-3	No	120	24

**Scheme 1:**
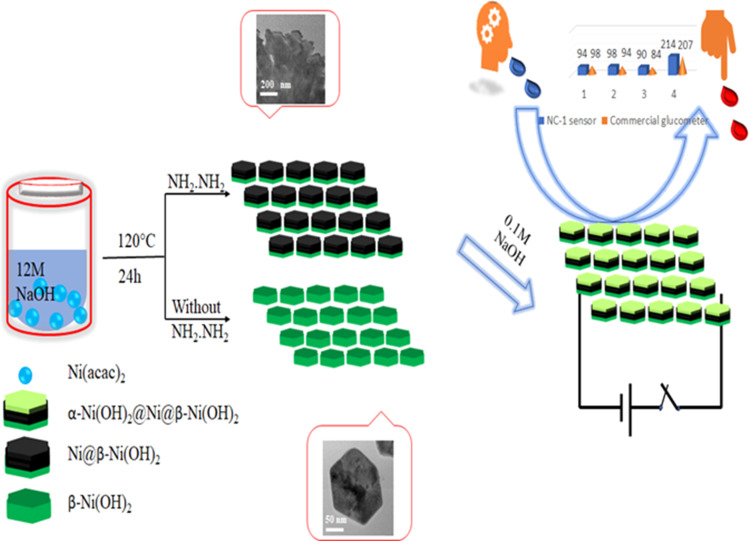
Fabrication and electrochemical performance of NC-1.

We attempted to alter the morphology by carrying out the reaction by varying the concentration of NaOH in the synthesis medium. The XRD pattern of the solid obtained in 1 M NaOH, presented in Fig. S2,† corresponds to β-Ni(OH)_2_. Similar reactions in the presence of hydrazine hydrate with 1 M NaOH failed to reduce β-Ni(OH)_2_ to nickel as indicated by the XRD patterns of these compounds shown in Fig. S2.† Based on microstructural analyses, we propose following mechanism for the structural and morphological evolution of (NC-3) β-Ni(OH)_2_ and *in situ* reduction of hydroxide to produce (NC-1) Ni@β-Ni(OH)_2_ nanomaterials: The reaction of nickel acetylacetonate with 12 M NaOH yields hexagonal sheets of β-Ni(OH)_2_. Subsequently, introduction of hydrazine hydrate reduces β-Ni(OH)_2_ to metallic nickel. Notably, this reduction process occurs effectively in the presence of a very high molar concentration of NaOH (<6 M), suggesting a base-catalyzed reduction mechanism taking place on the surface of nickel hydroxide hexagonal sheets. The extent of reduction is influenced by the reaction duration, with a 24 hours reaction yielding more nickel than a 12 hour reaction. Prolonged reaction times also lead to the agglomeration of smaller sheets, resulting in larger sheets with a composite Ni@β-Ni(OH)_2_ structure. Scheme 1 illustrates the synthesis conditions for the growth of hetero-nanostructures and electrochemical sensing properties of our proposed nano sensor.

### Electrochemical measurements

**Fig. 4:**
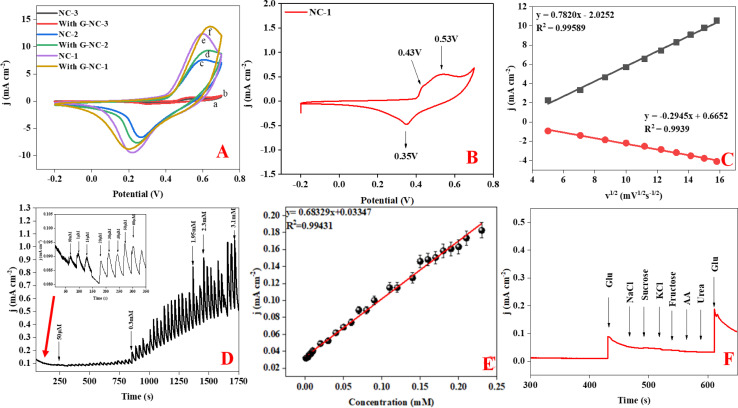
(A) CV of NC-1, NC-2 and NC-3 with and without glucose in the presence of 0.1 M NaOH. (B) CV graph of NC-1 at a scan rate of 50 mV s^−1^ in 0.1 M NaOH. (C) Linear relationship of anodic and cathodic peak current of NC-1 *vs. V*^1/2^. (D) Chronoamperometry of NC-1 with various concentrations of glucose at 0.45 V. (E) Linear relationship of NC-1 with respective glucose concentration (1 μM to 3.1 mM). (F) Amperometry response of NC-1 with other potential interfering compounds.

Following the initial microstructural analysis, all three compounds were characterized with cyclic voltammetry covering a voltage range from −0.2 to 0.8 V with the scan rate of 50 mV s^−1^, chronoamperometry, and interference studies in a 0.1 M NaOH electrolyte. Cyclic voltammetry (CV) graphs in Fig. 4(A) represent the current response of NC-1, NC-2, and NC-3 to potential sweep, in 0.1 M electrolyte and in the presence of 0.5 mM glucose. Upon addition of glucose, NC-3 exhibited no appreciable change in current response, owing to its poor conductivity and negligible electrocatalytic effect. Conversely, NC-1 and NC-2 displayed a relatable increase in current density with glucose addition. The emergence of an oxidation current and a discrete development of reduction current demonstrate good electrochemical catalytic ability of these compounds. Upon addition of glucose, NC-1 exhibited higher current density variation when compared with the response of NC-2. Furthermore, the difference between the anodic peak current with and without glucose is more pronounced in NC-1 than NC-2. This observation suggests that the NC-1, with its higher Ni/β-Ni(OH)_2_ ratio than NC-2, possesses better sensing property in comparison to NC-2. To understand the redox property of our heterostructures, detailed CV analysis of all the samples were carried out by varying the CV parameters. The CV graph of NC-1, as depicted in Fig. 4(B) exhibits dual anodic oxidation peaks at 0.43 V and 0.53 V, along with one cathodic peak at 0.35 V. Thus, we observe oxidation of two nickel species while only one type of nickel species is getting reduced. According to a report by Tong G. *et al.*,^41^ the anodic oxidation peak at 0.43 V can be attributed to the transformation of α-Ni(OH)_2_ to γ-NiOOH and the anodic peak at 0.53 V corresponds to the oxidation of β-Ni(OH)_2_ to β-NiO(OH).^8,41,56^ In a reported procedure by Ghanem *et al.*,^57^ a reduction potential peak in the range of −0.4 to −0.1 V is associated with the electrochemical reduction of γ-NiOOH back to Ni. However, we do not observe a reduction peak in the expected range. From this observation, we conclude that there is no regeneration of metallic nickel and 0.35 V reduction peak does not belong to reduction of γ-NiOOH to Ni. Tong G. *et al.*^41^ reported that a difference, (*E*_O_ − *E*_R_), of 0.15 V was observed between oxidation peak (*E*_O_) and reduction peak (*E*_R_) for α-Ni(OH)_2_/γ-NiOOH redox pair. In our system, we observe potential difference of 0.08 V (*E*_O_ − *E*_R_ = 0.43 − 0.35), which differs from the reported value.^41^ Therefore, if we consider the first oxidation peak as oxidation of α-Ni(OH)_2_ to γ-NiOOH, it becomes evident that we do not observe a cathodic peak equivalent to the reduction of γ-NiOOH back to α-Ni(OH)_2_. Furthermore, as shown in Fig. S3,† during oxidation of glucose, the anodic peak at 0.53 V shifted to a higher potential, while the cathodic peak shift was negligible. This anodic peak shift indicates the phase transition of α- to β-Ni(OH)_2_ and an increase in Ni(ii)/Ni(iii) active sites.^41^ Also after a continuous run of 350 cycles, the anodic oxidation peak of α-Ni(OH)_2_ disappeared, indicating depletion of the disordered hydroxide layer with aging in the electrolyte (Fig. S4†). From the above two observations, we have assigned the single cathodic peak at 0.35 V to the reduction of β-NiO(OH) to β-Ni(OH)_2_.^41,57^

To assess the glucose sensing capability of Ni@Ni(OH)_2_ heterostructure, CV analysis was carried out with standard additions of glucose. In Fig. S3,† we show the CV of NC-1 at different concentrations of glucose. The sequential addition of glucose increases the current output. Our experiments reveal that glucose detection range for Ni@Ni(OH)_2_ heterostructure, spans from 10 μM to 1 mM. Fig. S5† shows the current response of NC-2 with varying concentration of glucose. From the graphs, it is clear that NC-1 has better response with extended glucose detection range than NC-2. In NC-2, the relative number of active nickel sites is lower than that of NC-1 resulting in an expected reduction in the detection range from 10 μM to 1 mM to 10 μM to 0.25 mM. Fig. 4(C) displays the linear relationship obtained between both anodic and cathodic peak currents against *V*^1/2^ (square root of scan rate). CV curves of NC-1 at various scan rates in a 0.1 M NaOH electrolyte solution containing 0.5 mM glucose shown in Fig. S6.† The increase in current density with scan rate and the linear relationship shown in Fig. 4(C) suggests that the glucose oxidation follows a diffusion-controlled mechanism.^4^ The LOD, can be calculated using the equation (LOD = 3*σ*/*S*), where *σ* represents the standard deviation before the addition of analyte and *S* is the slope of the calibration graph. The ratio of slope to electrode area corresponds to the sensitivity of the electrocatalyst. Chronoamperometric studies were carried out for NC-1 at a peak potential of 0.45 V. Glucose was incrementally added at fixed intervals, and the current response was measured in a 0.1 M NaOH solution. The current signals improved with the addition of glucose (Fig. 4(D)). As depicted in Fig. 4(E), NC-1 exhibits a sensitivity of 683 μA mM^−1^ cm^−2^ in the 1 μM to 3.1 mM concentration range, accompanied by a low detection limit of 9.1 μM.

**Fig. 5:**
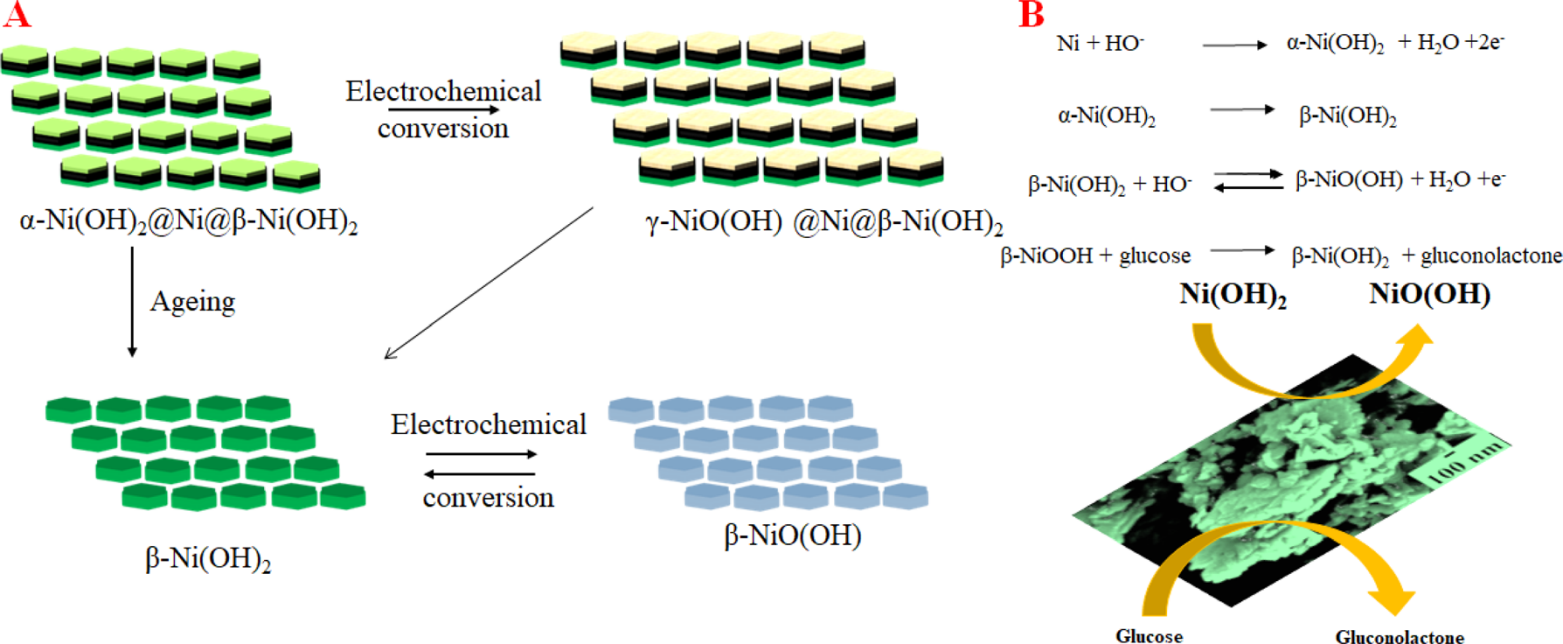
(A) The electrochemical pathway for NC-1 in 0.1 M NaOH. (B) Sensing mechanism of NC-1.

Based on the electrochemical analysis of our system in conjunction with existing literature, we propose the following mechanism happening at the interface of NC-1 nanosheets which is responsible for highly sensitive glucose detection. When NC-1 is immersed in 0.1 M NaOH electrolyte, applying a positive voltage results in the formation of an ultrathin layer of α-Ni(OH)_2_ on metallic nickel active sites. This newly formed disordered hydroxide layer participates in two competitive reactions:^58,59^ the oxidation of α-Ni(OH)_2_ to γ-NiOOH and the phase transformation of α-Ni(OH)_2_ to β-Ni(OH)_2_ in the presence of alkali medium. Additionally, the oxidized product, γ-NiOOH, undergoes conversion to β-Ni(OH)_2_, as there is a tendency for turbostratic nickel hydroxides to transition from a disordered to an ordered phase, in the presence of an electrolyte. Throughout the CV measurements, the formation of β-Ni(OH)_2_*via* these two pathways appears to be a dynamic process. Simultaneously, at any given point in time, the electroactive β-Ni(OH)_2_ maintains an ultrathin diameter due to the dynamic nature of the formation process. This high electrochemical surface area of β-Ni(OH)_2_ generated by dynamic phase change of α-Ni(OH)_2_ imparts a glucose oxidase enzyme like property to our Ni@β-Ni(OH)_2_ hetronanotructure. The metallic nickel active sites also provide the enhanced conductivity required for the sensor. This is evident from the Nyquist plot of the as synthesized compounds shown in Fig. S7.† NC-1 shows the lowest resistance followed by NC-2 and lastly NC-3 which has the highest resistance of the lot. The low resistance combined with high electrochemical surface area of our Ni@β-Ni(OH)_2_ nanozyme leads to ultra-high activity towards glucose sensing at low concentrations. Electrochemical dynamic change between different phases of nickel hydroxides in our system is pictorially illustrated by a modified Bode diagram in Fig. 5(A). Fig. 5(A) and (B) and Scheme 1 illustrates the mechanism of formation and electrochemical performance of the proposed glucose sensor developed in this study.

The remarkable sensitivity of NC-1 could be attributed to the formation of an ultrathin α-Ni(OH)_2_ surface layer on the nanosheet structure of highly active nickel sites. The subsequent conversion of alpha to ultrathin electrochemically active β-Ni(OH)_2_ occurs in 0.1 M NaOH. For fabricating a highly sensitive surface, generation of ultrathin layer of hydroxide electrochemical layer seems to be a crucial step. Thickness of the hydroxide layer formed on nickel sheet has a strong dependence on the concentration of electrolyte.^31^ To further validate our hypothesis, glucose sensing reactions were carried out by varying NaOH concentration. As the molarity of NaOH rises from 0.1 to 0.25 M, there is a significant rise in current density at sensing potential (Fig. S8†). However, it is noteworthy that upon addition of 0.5 mM glucose solution, current density decreased implying that an electrolyte concentration of 0.25 M is not as good as 0.1 M for sensing. This inverse relation between sensitivity and NaOH concentration supports our hypothesis that the thickness of *in situ* generated nickel hydroxide is directly proportional to the sensitivity, especially in lower concentration ranges of glucose.

To demonstrate the capacity of NC-1 as a non-enzymatic glucose sensor, it's crucial not only to demonstrate sensitivity but also to showcase selectivity for glucose in the presence of potential biological interference compounds such as sodium chloride (NaCl), urea, sucrose, and ascorbic acid (AA). The nickel hydroxide-based sensor exhibits excellent selectivity for glucose, with a glucose-to-interference ratio of 1 : 2 at the applied potential of 0.45 V (Fig. 4(F)). Furthermore, the stability of our proposed sensor was assessed by monitoring the current output signal of NC-1 in the presence of 0.5 mM glucose. Fig. S9† illustrates that NC-1 maintains its sensitivity even after 20 h of continuous amperometry analysis. The inset in Fig. S9† is the current response of the electrode upon addition of glucose at 300 seconds. Following this, the electrode was kept in the 0.1 M NaOH solution overnight. A continuous amperometry study showed that the current response was constant. After 60 000 seconds, we again added glucose to check the response and sensitivity of the electrode. From the Fig. S9,† it is evident that proposed sensor has excellent stability with respect to catalytic activity. We also deduced the response time of sensor to be ≈0.1 second. With these sought after qualities, commendable stability and response time, our sensor can be a viable material for fabrication of glucose sensor.

In diabatic patients, concentration of glucose varies in different bodily fluids including blood, saliva and sweat. For instance, concentration of glucose in saliva for a normal healthy individual will be well below 0.08 mM whereas for a diabatic patient the levels often range between 0.2 mM to 0.4 mM.^60,61^ A good salivary sensor should not only have a good sensitivity in this preferred range but should also possess a lower LOD such that normal salivary glucose levels could also be detected. Since the linear detection range of Ni@β-Ni(OH)_2_ nanozyme encompasses the salivary glucose concentrations, our sensor could be a reliable tool for detecting glucose in saliva, given its high responsiveness within this desired glucose concentration range.

To demonstrate the practical application of NC-1 as a commercial salivary glucose sensor under authentic biological conditions, we collected saliva and blood samples from four volunteers strictly following ethical protocol (IEC Protocol No. IITH/IEC/2023/12/26).^62^ Of the four volunteers, three are regarded as normal subjects, and the fourth is considered to be prediabetic. Employing a market-available glucometer, glucose concentrations in blood of four participants were measured using commercial test strips. Corresponding salivary glucose levels of the volunteers were calculated from their blood glucose values using a regression relation from literature. The formula used for calculating random blood glucose is [serum glucose (mg dL^−1^)] = 95.607 + 27.710 [saliva glucose (mg dL^−1^)] and fasting blood glucose is [serum glucose (mg dL^−1^)] = 68.650 + 28.050 [saliva glucose (mg dL^−1^)].^63^ The salivary glucose levels thus calculated are given as reference levels in Table 2.

**Table 2:** Comparison of saliva glucose concentrations from blood glucose levels with the valued measured by NC-1 in this study

Volunteers	Blood glucose level (mg dL^−1^)	Type	Blood glucose level in mM	Salivary glucose level from BG (μM)	Salivary glucose level from NC-1 sensor
1	98	FBS	5.43	58	49
2	94	FBS	5.2176	55	59
3	84	FBS	4.6625	30	38
4	207	RBS	11.48	223	237

**Fig. 6:**
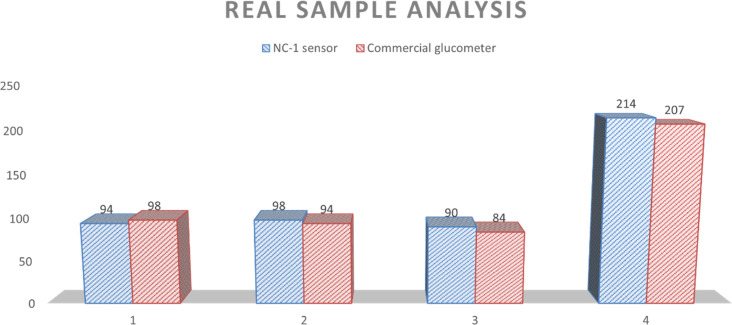
Comparison of blood glucose level calculated from NC-1 sensor and commercial glucometer.

NC-1 nanozyme was used to measure salivary glucose levels of volunteers by standard addition method. For this process, known concentrations of glucose were added to human salivary samples and amperometric studies were performed to measure the salivary glucose levels. In Fig. (6), salivary glucose levels calculated from blood glucose levels can be compared with the measured salivary glucose in this study. For our prediabetic volunteer, for a blood glucose level of 11.5 mM, calculated salivary glucose level is 223 μM. Salivary glucose levels measured in the saliva of prediabetic volunteer by our NC-1 is 237 μM. Similarly, for our healthy volunteers, blood glucose concentration varied from 4.7 mM (volunteer 1), 5.2 mM (volunteer 2) to 5.4 mM (volunteer 3). The salivary glucose levels detected by our sensor for these volunteers are 38 μM (volunteer 1), 59 μM (volunteer 2) and 49 μM (volunteer 3). To check the sensitivity of the non-enzymatic sensor in extreme conditions, we tested the random blood glucose/salivary glucose for our prediabetic volunteer and fasting salivary glucose concentrations for our healthy volunteers. The rationale behind this is as follows. For a healthy normal individual, fasting blood glucose will be the lowest measured value in a day. For a prediabetic/diabatic volunteer, random blood glucose level will most probably be higher than the fasting blood sugar values. If the sensor shows an ability to measure across these two values, it establishes the versatility of the sensor in terms of usage. From Fig. (6), it is evident that there is excellent agreement between reference salivary glucose levels and the results of salivary glucose concentrations measured by Ni@β-Ni(OH)_2_ nanozyme based sensor. From this real sample analysis studies, we can see that saliva glucose levels can be correlated to blood glucose levels. Similar to blood glucose level, salivary glucose levels also change depending on the health conditions and sample collection time. Salivary glucose sensors can thus be reliably used for non-invasive glucose detection and point-of-care tests.

In Table S1.† ^64–66^ we can compare the proposed non-enzymatic glucose sensor with other reported Ni@Ni(OH)_2_ based sensors. The salivary glucose levels vary between 0.02 mM and 0.4 mM. Our proposed glucose sensor with linear detection limit from 0.001 mM to 3.1 mM. The table clearly demonstrates that our sensor is viable in its ability to sense lower concentrations of glucose. Several reported Ni(OH)_2_ sensors in Table S1† exhibits high sensitivity like 3200 μA mM^−1^ cm^−2^ for nickel hydroxide grown on nitrogen-doped nanodiamond, 2761 μA mM^−1^ cm^−2^ for nickel hydroxide on three-dimensional porous nickel template, 2366 μA mM^−1^ cm^−2^ for Ni(OH)_2_ grown on three-dimensional graphene foam and 1942 μA mM^−1^ cm^−2^ for Ni(OH)_2_ grown on carbon cloth. The higher sensitivities of these sensors could be associated with the fact that the sensing Ni(OH)_2_ nanostructures are grown on highly conducting substrates like graphene foam, porous nickel foam, carbon cloth or nanodiamond. The porous conducting substrates will enable transfer of electron from α-Ni(OH)_2_ sensor to electrode very efficiently. So, it is not unexpected for these sensors to exhibit high sensitivity. But in our Ni@β-Ni(OH)_2_ nanozyme, the electroactive layer is a free-standing turbostratic hydroxide formed on a nickel surface by immersion into NaOH electrolyte. Without the aid of any conducting substrates, the sensor proposed in this work exhibits a sensitivity comparable to that of α-Ni(OH)_2_ sensors grown on substrates which are complex and/or difficult to fabricate.

**Fig. 7:**
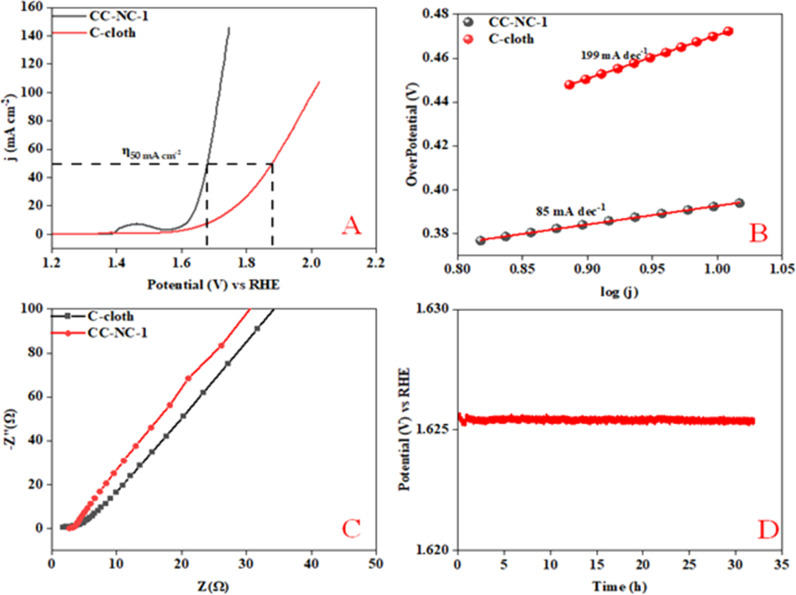
(A) Polarization curve of NC-1, (B) Tafel plot, (C) electrochemical impedance spectroscopy, (D) chronoamperometry of NC-1 loaded on carbon cloth @ 50 mA cm^−2^.

**Fig. 8:**
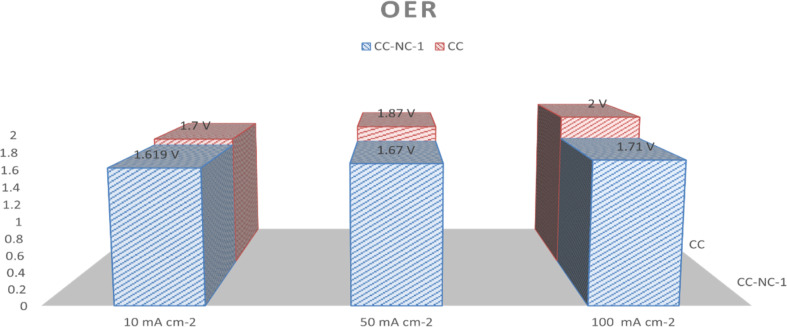
Simplified OER comparison of CC/NC-1 with CC at various current densities.

**Fig. 9:**
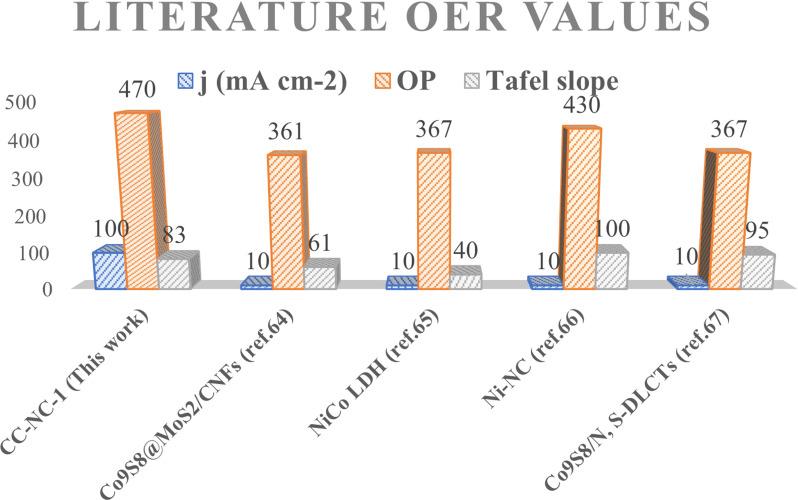
Literature OER comparison of CC/NC-1 with various catalysts.

Nickel hydroxide is also very well known for its electrocatalytic efficiency to split water, to be precise in oxygen evolution reaction. This catalytic property of Ni(OH)_2_ could potentially interfere with non-enzymatic glucose sensing process often resulting in an over estimation of glucose. To find out the probability of the presence and/or extent of this unwanted side reaction in glucose sensing ability, we carried out electrochemical water splitting of NC-1. For this purpose, conducting ink prepared by mixing NC-1, Nafion and solvent was drop-casted on a glassy carbon electrode (GCE). When electrochemical OER studies were carried out in 1 M KOH, no oxygen evolution reaction was observed in the expected potential window. On the other hand, NC-1 loaded onto carbon cloth by *in situ* growth exhibited reasonable electrocatalytic efficiency in OER. In Fig. 7(A), iR-corrected LSV curve in 1 M KOH solution with a scan rate of 5 mV s^−1^ is shown. NC-1/CC exhibits an overpotential of 1.67 V at 50 mA cm^−2^ current density. The Tafel plot value is 83 mV dec^−1^ as shown in Fig. 7(B). We have consolidated the OER activity of NC-1/CC in Fig. 8. To understand the reason behind the emergence of OER activity exclusively by NC-1 grown on carbon cloth, we carried out impedance analysis as shown in Fig. 7(C). From the graph, it is evident that the introduction of carbon cloth has reduced the resistance of NC-1 and in turn increased the conductance resulting in OER activity of NC-1/CC. To further assess the stability of catalyst for OER activity, chronoamperometry measurements (Fig. 7(D)), were conducted at 10 mA cm^−2^. NC-1/CC electrocatalyst exhibited excellent stability for over a period of 32 h, without any loss of activity. This OER efficiency is comparable to reported works by other research groups. In Fig. 9, we can compare the OER efficiency of CC/NC-1 with other reported Ni(OH)_2_ systems.^67–70^ On the contrary, we do not observe any OER activity for NC-1 drop-casted onto GCE. Thus NC-1 powder, a hetero nanostructure, formed by one-pot solvothermal method can function as a commendable non-enzymatic salivary glucose sensor without any interference from OER current generation. This unique and much sought-after ability of Ni@Ni(OH)_2_ in powder form, makes it an excellent choice for sensor-based applications.

## Conclusion

In summary, we have successfully synthesized a surfactant-free Ni@Ni(OH)_2_ glucose sensor through a low-temperature solvothermal method. This nickel-based sensor exhibits remarkable sensitivity, with an impressive value of 683 μA mM^−1^ cm^−2^ within the glucose concentration range of 1 μM to 3.1 mM, while also demonstrating a low detection limit of 9.1 μM. The outstanding performance of this nanozyme sensor can be attributed to the formation of an ultrathin nickel hydroxide electrochemical surface. With its high sensitivity in detecting low glucose concentrations and material selectivity, our proposed nanozyme sensor holds significant promise as a non-enzymatic, non-invasive glucose sensor for point-of-care applications.

## Experimental section

All commercial reagents were purchased and used as received, without further refinement. Nickel(ii) nitrate hexahydrate (Ni(NO_3_)_2_·6H_2_O), and hydrazine hydrate reagent grade (N_2_H_4_ 50–60%), were purchased from Sigma Aldrich. Acetyl acetone (99.5%) was purchased from Finar. Sodium hydroxide, potassium hydroxide, ammonia solution (25%), d-glucose, urea, ascorbic acid, sucrose, fructose and sodium chloride were procured from SDFCL. Nafion solution was acquired from Sigma Aldrich.

Nickel(ii) acetylacetonate hydrate was prepared following a reported procedure with slight modifications.^49^ Nickel(ii) acetylacetonate hydrate (0.2 g, 0.5 mmol) was dispersed in NaOH (15 mL, 12 M) and hydrazine hydrate (300 μL, 9.6 mmol) by stirring the mixture at ambient temperature for 15 min. The solution was later placed in a 21 mL Teflon-lined autoclave and thermally reacted at 120 °C for 24 hours. Once the autoclave was cooled to room temperature, products obtained were washed with isopropanol and methanol. The washed product was separated under centrifugation (4500 rpm) and dried in a desiccator under vacuum. Table 1 shows the synthesis conditions of compounds prepared, analysed, and studied in this report.

### Material characterization

X-ray diffraction (XRD) analysis was conducted using Rigaku D/max-B instrument with Cu Kα radiation to identify the crystal structures of as prepared compounds. For surface morphology analysis FESEM (Merlin compact) was employed. Vibrational studies were carried out with FTIR (Thermo-Fischer) and Raman spectroscopy (Horiba). The surface elemental characterization has been performed with XPS (Axis supra).

### Electrochemical measurements

Electrochemical characterization of the as-synthesized compounds was tested using KLyte and AUTOLAB (PGSTAT204) electrochemical workstation. As prepared compound (5 mg) was dissolved in 300 μL of water and 20 μL of Nafion binder solution. The compound was sonicated for 15 min to form an ink and 10 μL of the ink was drop-casted onto the glassy carbon electrode (GCE). The deposition was carried out such that the amount of electrocatalyst was maintained as 0.16 mg cm^−2^. Platinum wire and Ag/AgCl electrodes were used as counter and reference electrodes respectively throughout the analysis. 0.1 M NaOH was used as an electrolyte solution for glucose sensing.

The electrochemical oxygen evolution reaction (OER) catalyst was prepared *via in situ* synthesis of catalyst on pretreated 1 × 1 carbon cloth as a working electrode. 1 M KOH was employed as an electrolyte solution. The linear sweep voltammetry (LSV) was carried out at 5 mV s^−1^ scan rate and the LSV graphs were corrected with iR-correction (100%) to avoid electrolyte resistance. The electrochemical impedance spectroscopy (EIS) measurements were carried out from the frequency range of 100 MHz to 0.01 Hz with an amplitude of 10 mV. All the electrochemical measurements were carried out at room temperature.

## Data availability

The data supporting this article have been included as part of the ESI.†

## Author contributions

Gomathi: conceived the project, resources, data curation, visualization, writing – review & editing, supervision. C. Subrahmanyam: resources, supervision. Priya Pathmanathan: methodology, investigation, data curation, validation, writing – original draft, visualization, experimentation. Asha Ramesh: resources, real sample analysis.

## Conflicts of interest

There are no conflicts to declare.

## Supplementary Material

RA-014-D4RA03559C-s001
