# Supplementary material for: In situ generation of turbostratic nickel hydroxide as a nanozyme for salivary glucose sensor
Source: RSC Adv. 2024 Jul 9;14(30):21808–20. doi: 10.1039/d4ra03559c (PMC11232413; doi:10.1039/d4ra03559c)
Supplement: RA-014-D4RA03559C-s001 [file RA-014-D4RA03559C-s001.pdf]

Electronic Supplementary Material (ESI) for RSC Advances.
This journal is © The Royal Society of Chemistry 2024

SUPPORTING INFORMATION

The supporting information includes FESEM images, XRD patterns, electrochemical measurements, and stability studies of the compounds prepared in this study.

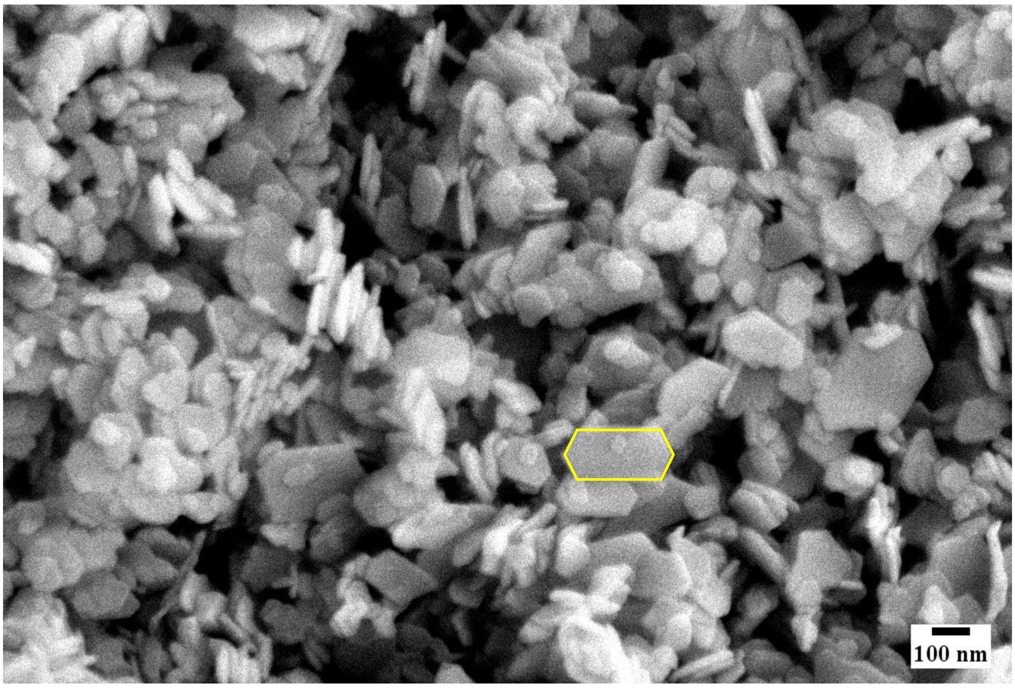

Figure S1. FESEM image of NC-2.

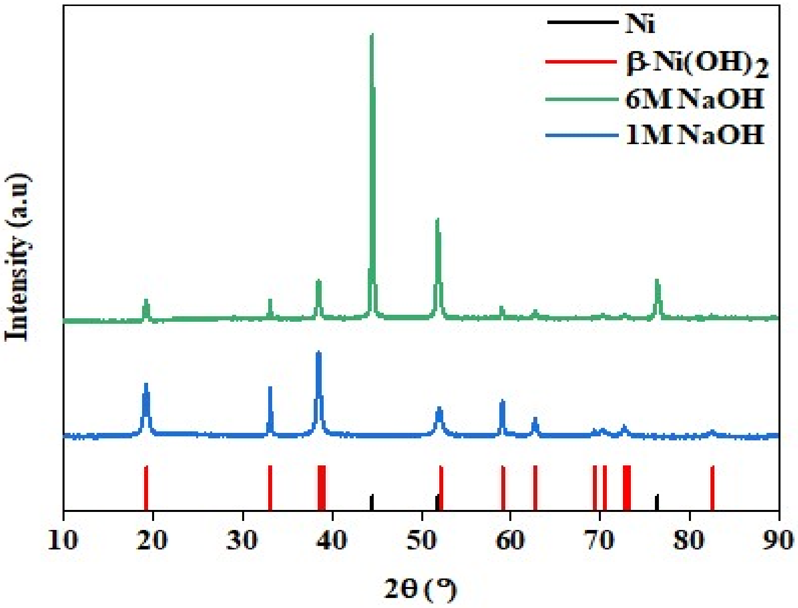

Figure S2. Concentration variation of NaOH for the synthesis of Ni@Ni(OH)$_2$.

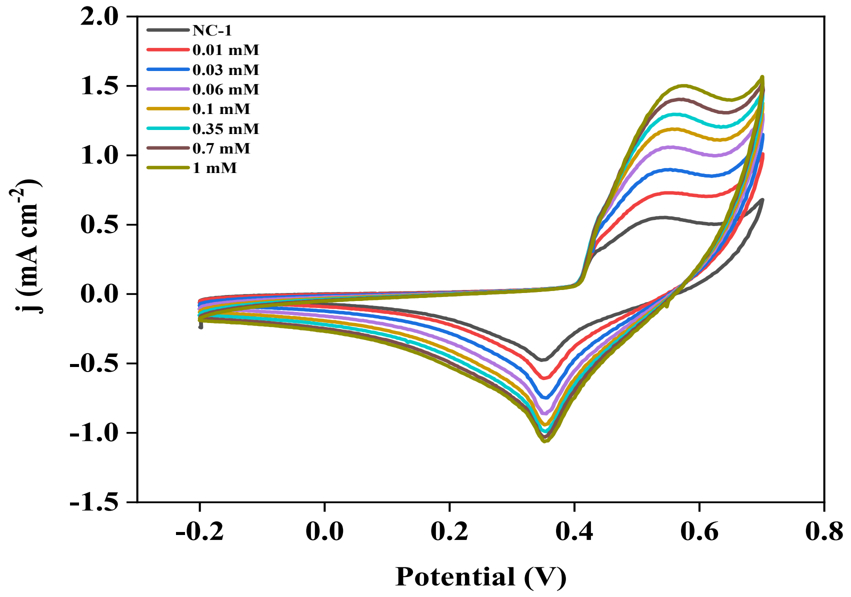

Figure S3. CV of NC-1 with various concentrations of glucose at the scan rate of 50mV s^{-1}.

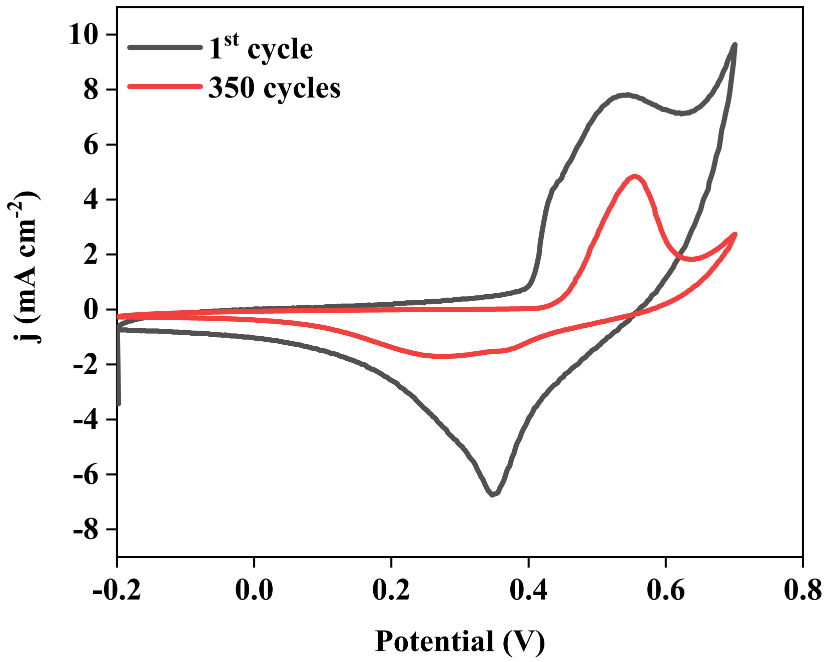

Figure S4. Number of cycles NC-1 from α to β- $Ni(OH)_2$.

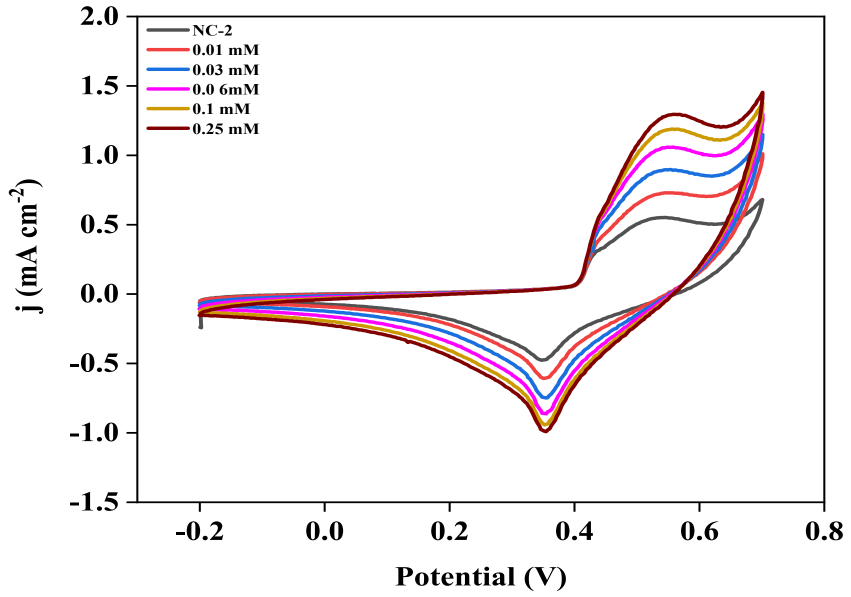

Figure S5. Concentration variation of NC-2 with the addition of glucose (both phases).

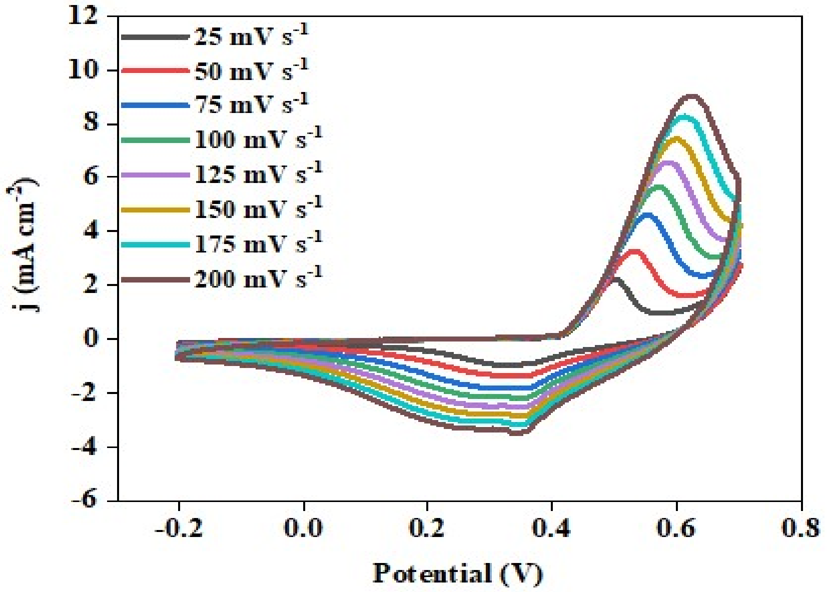

Figure S6. Scan rate variation of NC-1 with 0.5 mM glucose in 0.1 M NaOH.

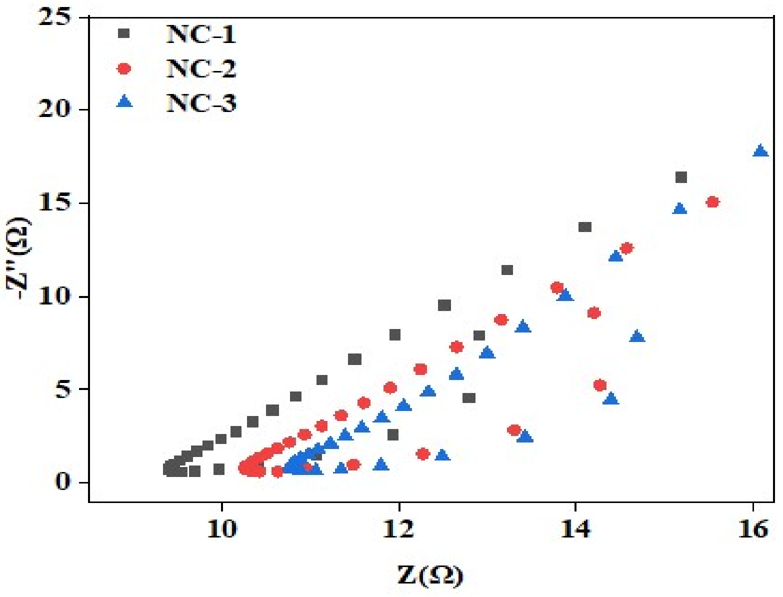

Figure S7. Nyquist plot of as-synthesized compounds with and without glucose addition.

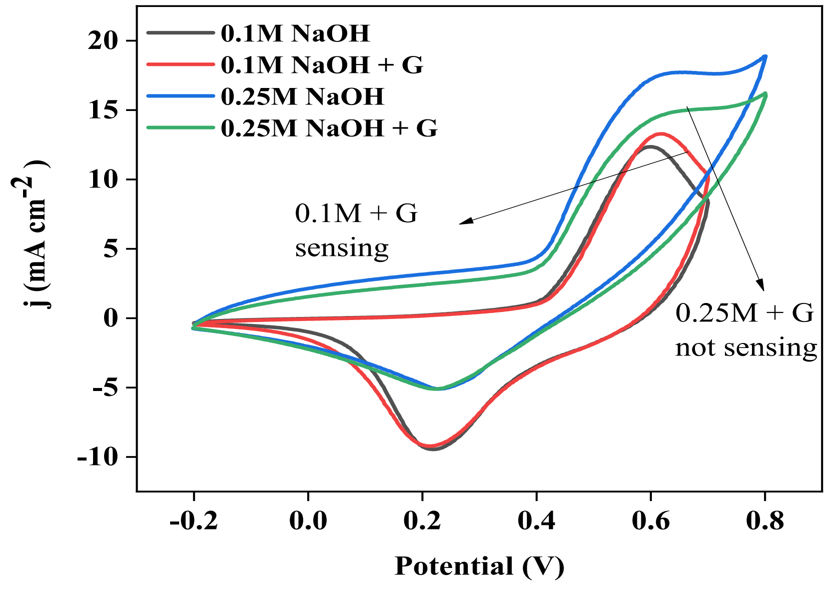

Figure S8. Concentration variation of NaOH in the presence of NC-1 with and without glucose.

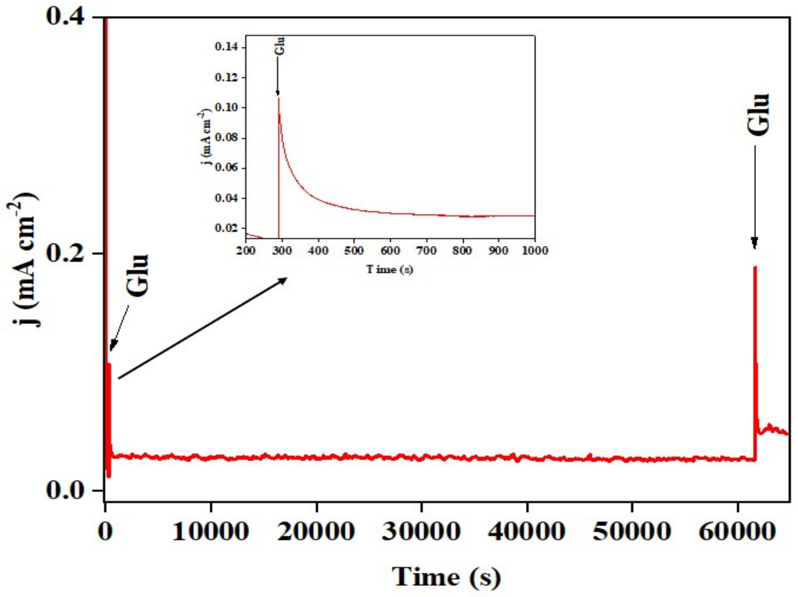

Figure S9. Response of NC-1 with the addition of glucose.

Table S1: Comparison of salivary glucose sensors based on $Ni(OH)_2$ based nanocomposites

S.No	Electrode	Linear range	Detection limit(µM)	Sensitivity (µA mM^{-1} cm^{-2})	Reference
1	$Ni/Ni(OH)_2$	1µM – 3.1mM	9	683	This work
2	$Ni(OH)_2$&NiOOH film/Ni	1µM – 1.3mM	0.414	5.7584	[31]
3	β-$Ni(OH)_2$/NP-Ni/MG	Upto 18mM	0.35	1496	[48]
4	$Ni(OH)_2$/CC electrode	4µM – 0.6mM	0.47	1942	[29]
5	α-$Ni(OH)_2$/rGO/NF	0.5 mM – 22.5 mM	0	95.5	[47]
6	$Ni(OH)_2$/3DPN templates	0.46 µM – 2.1 mM	0.46	2761.1	[68]
7	$Ni(OH)_2$/NND film	20 µM – 1 mM; 1-9 mM	1.2	3200,1410	[69]
8	$Ni(OH)_2$/ITO; NiO/ITO	0.1 mM–156 mM; 0.01-30 mM	70; 8.1	12.09; 24	[44]

9	CuO/FTO	5 µM – 0.225 mM	0.41	3072	[70]
